# Landmark-Assisted Compensation of User’s Body Shadowing on RSSI for Improved Indoor Localisation with Chest-Mounted Wearable Device

**DOI:** 10.3390/s21165405

**Published:** 2021-08-10

**Authors:** Md Abdulla Al Mamun, David Vera Anaya, Fan Wu, Mehmet Rasit Yuce

**Affiliations:** Department of Electrical and Computer Systems Engineering, Clayton Campus, Monash University, Melbourne, VIC 3800, Australia; md.mamun1@monash.edu (M.A.A.M.); david.veraanaya@monash.edu (D.V.A.); fan.wu@monash.edu (F.W.)

**Keywords:** indoor localisation, fingerprinting, landmark, wearable device, inertial measurement device, motion mode detection, body shadowing compensation, nearest neighbour

## Abstract

Nowadays, location awareness becomes the key to numerous Internet of Things (IoT) applications. Among the various methods for indoor localisation, received signal strength indicator (RSSI)-based fingerprinting attracts massive attention. However, the RSSI fingerprinting method is susceptible to lower accuracies because of the disturbance triggered by various factors from the indoors that influence the link quality of radio signals. Localisation using body-mounted wearable devices introduces an additional source of error when calculating the RSSI, leading to the deterioration of localisation performance. The broad aim of this study is to mitigate the user’s body shadowing effect on RSSI to improve localisation accuracy. Firstly, this study examines the effect of the user’s body on RSSI. Then, an angle estimation method is proposed by leveraging the concept of landmark. For precise identification of landmarks, an inertial measurement unit (IMU)-aided decision tree-based motion mode classifier is implemented. After that, a compensation model is proposed to correct the RSSI. Finally, the unknown location is estimated using the nearest neighbour method. Results demonstrated that the proposed system can significantly improve the localisation accuracy, where a median localisation accuracy of 1.46 m is achieved after compensating the body effect, which is 2.68 m before the compensation using the classical K-nearest neighbour method. Moreover, the proposed system noticeably outperformed others when comparing its performance with two other related works. The median accuracy is further improved to 0.74 m by applying a proposed weighted K-nearest neighbour algorithm.

## 1. Introduction

Knowledge about location information becomes the key to numerous location-based services (LBS) in various application domains including healthcare and safety, search and rescue, assisted living, robotics, shopping and museum assistance, context awareness and social networking, advertising, and marketing [1]. One of the key prerequisites to successfully empower these services is estimating the position of a subject of interest. This task can be effortlessly accomplished by employing the receivers of the Global Navigation Satellite System (GNSS) with direct line-of-sight (LOS) scenarios in the case of outdoors. The existence of the complex nature of indoors in terms of geometrical structures, presence of numerous objects made of multivariate materials, and the variations in ambient meteorological conditions lead to the reflection, refraction, or even complete blockage of the GNNS signal. Hence, the GNSS is unable to produce the desired accuracy required for the indoors [2].

Typically, an indoor localisation system utilises an infrastructure inside a building with a set of devices wirelessly connected to locate an unknown target carrying devices compatible with that network. Various technologies are used so far for indoor localisation including Bluetooth low energy (BLE), radio frequency identification (RFID), ultra-wideband (UWB), ultrasound, wireless local area network (WLAN), and wireless sensor network (WSN) [1]. Among them, RSSI-based WSN technology has drawn massive attention of the researchers owing to the emerging usability for numerous IoT applications as well as the easiness in RSSI acquisition. RSSI is the standard to measure the received signal power, which is used by various methods, such as propagation modelling, trilateration, multidimensional scaling, DV-Hop, and fingerprinting for location estimation [1]. From them, the RSSI fingerprinting approach offers satisfactory results without the requirement of additional costs in terms of hardware and computation. The fingerprinting method comprises two major phases: the offline training phase and the online localisation phase. The training phase builds a database, named the radio map, by gathering geotagged RSSI fingerprint data from visible radio modules/anchor nodes, named reference nodes (RN), at known locations, named reference points (RP). The online phase calculates the position of an unknown target node by comparing a query fingerprint with the radio map.

Recently, WSN has become an attractive research area, especially for various monitoring applications, due to its real-time and accurate response, coverage, and simple infrastructure. With the continuous advancement and miniaturisation of sensing, as well as communication technologies, wearable devices are becoming an essential component for daily living. WSNs using wearable sensor devices are emerging for many IoT applications. Acquiring information about the location of a user is one of the key features of wearable devices, which becomes one of the major issues for WSN due to the presence of a massive number of wearable sensor nodes in modern IoT applications. 

One of the major limitations of RSSI-based indoor localisation is the erroneous determination of RSSI. The main reasons for this are the abovementioned complex nature of indoor environments and the non-line-of-sight (NLOS) situations triggered by the signal blockage between a sender and a receiver. In the case of wearable devices, the user’s body can introduce the NLOS scenario that leads to an additional effect on the resulted RSSI. The human body encompasses around 70% of water that can absorb part of the radio signal [3]. Moreover, the human body can scatter the longer radio signal waves while reflecting or attenuating the shorter ones due to its conductive nature [4]. Thus, the presence of the human body in between a sender and a receiver influences the propagation of radio signal that can cause an incorrect calculation of RSSI. Eventually, this circumstance leads to an erroneous position estimation in RSSI fingerprinting-based localisation when a wearable device calculates incorrect RSSI from multiple RNs. Researchers have already reported that human body shadowing could distort the RSSI by up to 5 dBm, causing a positioning performance degradation of about 67%, where there is a strong correlation between that distortion and user orientation [5]. Besides the NLOS scenarios created by the wearable user’s body, there may be other sources that can introduce errors in RSSI calculation, including the presence and movement of other humans, as well as objects in between the sender and receiver. Although it is impractical to characterise all such errors precisely due to the randomness in the numbers and sizes of those humans and objects, it is realistic to deal with the systematic source of error caused by the wearable user’s body [5]. In the case of RSSI fingerprint-based localisation, the user’s body shadowing effect (BSE) can be mitigated explicitly by modelling and compensating this systematic error when comparing a query RSSI fingerprint with a radio map. Although there are several studies that investigate the effects of user’s BSE on wireless signal transmission, there are still some challenges that require further attention, including:

- The estimation of the orientation angle between a user and an RN in real time.
- The derivation of a BSE compensation model that can mitigate the user’s body effects for every single orientation angle scenario instead of some discrete orientation angles.
- The adaptation of the orientation angle estimation and BSE mitigation methods in real-life indoor localisation applications.

Knowledge of the indoor area, i.e., the spatial information, may be an assistive tool that can be leveraged to improve the indoor localisation accuracy without paying extra cost for setup. Landmark, i.e., the sensory landmark, is one such piece of spatial information that is distributed naturally to a floor plan and can be helpful to enhance localisation accuracy [2,6]. Specifically, landmarks are the markers in the indoor map that experience specific signal patterns all the time when one or more sensors meet those markers. Although some previous works utilised landmarks for robot tracking or pedestrian dead reckoning (PDR)-based positioning, this work used landmarks as a supportive tool for mitigating the human BSE on RSSI.

The aim of this study is to compensate the user’s BSE on RSSI to improve the RSSI fingerprinting-based indoor localisation performance with a chest-mounted wearable device in a WSN setting. The proposed fingerprinting system composed the offline and online phases similar to the traditional fingerprint methods. However, the online phase performs several additional tasks to mitigate human body shadowing errors. To compensate the RSSIs of a query fingerprint with proper values, the angle between the wearable device and the RNs is estimated considering the user’s orientation. The concept of landmark graph along with arctangent function is utilised for angle estimation. To identify a landmark, an IMU-aided decision tree-based motion mode detection classifier is implemented. Then, a human body shadowing compensation model is proposed to correct the RSSIs of the query fingerprint. Finally, both the classical k-nearest neighbour (K-NN) and weighted k-nearest neighbour (WK-NN) algorithms are employed to calculate the location of the unknown target. The main contributions of this research are as follows:

- An in-depth analysis of the behaviour of XBee RSSI is performed to investigate its effect on the user’s body.
- A unique method is proposed to estimate the orientation angle between a user and the RNs.
- A new model is proposed that can compensate the user’s BSE on XBee RSSI for every possible orientation angle.
- A landmark-assisted weight calculation method is used to implement the WK-NN algorithm to improve the localisation accuracy.
- Experiments were conducted in a real indoor scenario by applying the proposed method and model for real-time application.

The remainder of the paper is organised as follows: Section 2 discusses and compares the existing literature related to this study; Section 3 presents an in-depth analysis of the effect of the user’s body on RSSI; Section 4 presents an overview of the proposed system; Section 5, Section 6 and Section 7 describe the details of the proposed system that includes landmark identification, user’s BSE compensation, and fingerprinting localisation, respectively; Section 8 discusses the experiments and illustrates the results by comparing with other related works; finally, Section 9 concludes this study with future recommendations.

## 2. Related Works

Until now, there are numerous studies that investigate the effects of the human body shadowing on wireless signal transmission to characterise and model the wireless channel and antenna radiation by focusing on various aspects of body-centric radio frequency-based communication. Moreover, human BSEs have been analysed and modelled for a variety of applications, including people counting [7,8], fall detection [9,10], and activity recognition [11], as well as proximity detection for coronavirus contact tracing application [12]. Although several studies have been performed on analysing the effect of human body shadowing on radio signal transmission targeting indoor localisation applications, they have mostly neglected the derivation of a compensation model and/or the integration of a compensation model to implement a real-time localisation system. Table 1 presents an overview of the existing literature that discusses human BSEs on wireless signal transmission for indoor localisation and tracking applications.

In the literature, researchers utilised several wireless technologies; this study only focuses on the systems that exploited RSSI as their measurement approach and/or fingerprinting as their localisation method. An RFID-based system is presented in [13], where the authors demonstrated the improvement in indoor localisation accuracy by compensating the errors caused by human body shadowing. Channel models for both the LOS and NLOS cases were derived, and RFID RSSI-based Monte Carlo localisation was implemented to achieve an accuracy of 1.18 m. However, this approach has very limited applicability for real-time location tracking applications because the differentiation between the LOS and NLOS conditions were assessed manually.

**Table 1:** Comparison among existing studies focused on human body shadowing effects on wireless signal for indoor localisation and tracking applications.

Research Type ^1^	Wireless Technology	Measurement Approach	Sensor Placement	Angle Variations (°)	Localisation Methods	Evaluation ^2^	Localisation Accuracy (m)	Ref.
An, Co	2.4 GHz RF	RSSI	Handheld	90	Fingerprinting	Ex	2.94 (50th percentile)	[5]
An, Co	WiFi	RSSI	Handheld	45	Fingerprinting	Ex	1.65 (average)	[14]
Mo, Co	RFID	RSSI	Wrist	N/A	Monte Carlo	Ex	1.18	[13]
An, Mo, Co	WiFi	RSSI	Chest, Handheld	N/A	NLLS	Ex	N/A	[15]
An, Mo, Co	Zigbee	RSSI	Chest, Back	45	Fingerprinting	Ex	2.5 (median)	[16]
An, Mo, Co	Zigbee	RSSI	Chest, Back, Wrist	45	Fingerprinting	Si and Ex	2.99 (50th percentile for chest)	[17]
An, Mo	WiFi	RSSI	Handheld	N/A	Fingerprinting	Ex	N/A	[18]
An, Co	WiFi	RSSI	Handheld	90	Fingerprinting	Ex	2.00 (50th percentile)	[19]
An, Mo, Co	BLE	RSSI	Handheld	3 orientations	Ranging, Trilateration	Ex	0.77 (mean)	[20]
An, Mo, Co	Zigbee	RSSI	Chest	15	Fingerprinting	Ex	0.74 (median)	This study

^1^ An = analysis; Mo = modelling; Co = compensation. ^2^ Ex = experiment; Si = simulation.

From Institute of Electrical and Electronic Engineers (IEEE) 802.11 family of standards, the impact of the human body on RSSI-based ranging measurements for cooperative localisation is presented in [15]. The authors investigated both the body and hand grip effects on RSSI among the neighbouring nodes. This study demonstrated that there is no significant improvement in cooperative localisation, compared to the noncooperative case, if the BSEs are not mitigated correctly. In [18], a mathematical model is proposed to mitigate the user’s BSE on RSSI of WiFi signals for improving indoor positioning accuracy. Handheld mobile devices were used to collect WiFi signals in a multipath-free environment, both for the LOS and NLOS cases, to analyse the BSEs. Finally, a model was derived that can intensify the strength of the signals which are coming through the NLOS states caused by the user body. Still, the authors did not discuss the methods of orientation estimation for real-time applicability of the proposed model. Moreover, this study only considers the handheld mobile devices for the experiment, and thus the model may not be compatible with body-attached wearable devices. In [5], the authors presented the first fingerprinting-based indoor localisation system that considered the subject’s BSEs on signal RSSI for position estimation. A radio map was created by using both the empirical measurements of RSS and a signal propagation model. To reduce the location estimation error that is cause by the user’s body, RSSI fingerprints were collected for four orientations of the subject’s body, compared to the RNs, in terms of four directions, i.e., north, south, east, or west. A K-NN search algorithm was employed, and a median accuracy of 2–3 m was achieved after compensating the human BSE. However, this solution only analyses the effect of user orientation on location estimation and falls short of proposing any compensation model with its real-time applicability to mitigate that effect. To solve the issue of estimating user orientation in real time, King et al. described an approach named COMPASS, where the authors utilised a digital compass to acquire the user’s orientation during both the offline and online phases [14]. During the offline phase, radio fingerprints were collected from each RN for eight orientations in every 45° angle position. In the online phase, a subset of fingerprints from the radio map was preselected based on the user orientation, and a probabilistic algorithm was applied to the subset to calculate the user position. Results demonstrated that considering the body orientation improved the localisation accuracy, where the average accuracy was 1.65 m. Yet, the radio map becomes highly redundant as eight radio fingerprints corresponding to eight directions, i.e., in every 45°, were collected for a single RP. As a result, the search space increases by eight times, which can cause an extra burden on the system performance in terms of computation cost and memory requirement for a large environmental area. It may even become infeasible for resource-constrained wearable devices for edge computing in the case of real-world applications. Moreover, it also increases the cost of the offline phase in terms of time and labour. Additionally, using COMPASS may produce high errors in orientation estimation for indoors, especially around the objects that have electromagnetic radiations. A similar approach was applied in [19], where the authors used mobile phone integrated compass and collected radio fingerprints for four orientations in the offline phase. During the online phase, they narrowed down the search space by applying a clustering method that used both the signal domain and spatial domain. An adaptive weighted K-NN algorithm was developed, which achieved an accuracy of 2.0 m for the 50th percentile; however, this study only considers four orientations of the human body in four directions, which is not enough to explore the complete variations of RSS values around a body.

From Zigbee-based indoor localisation solutions, a body-worn device is used in [16], where the authors analysed the BSEs on RSSI of 2.4 GHz ZigBee signals. Two tags were attached on the chest and back of a wearer, and data were collected at different angular positions. The arc tangent function is used for orientation estimation, and a simple cosine model is used to compensate the user’s BSE. Another improved version for BSE compensation in indoor localisation is proposed by the same group in [17], where the authors presented two solutions for improving localisation accuracy. In the first solution, a subject requires multiple wearable tags that need to be mounted in different positions to calculate RSSI. Then, their means are used as the input for fingerprint matching with reference fingerprints from a radio map generated using the WHIPP tool [21]. In the second solution, an arc tangent function is used to estimate the orientation of the target, where the target’s current location is calculated by averaging four previous positions. To mitigate the BSE, two compensation models are proposed: one is a basic over/underestimation model, and the other is a simulation-based three-dimensional model. Results demonstrated that the proposed models could compensate the BSE to improve localisation accuracy from 3.48 m to 2.99 m (50th percentile for chest). However, this approach requires a subject to wear multiple tags, which may limit its scope for real-world application. Moreover, as the radio map created during the offline phase did not consider the BSE, the estimated location accuracy will be low and, eventually, the orientation estimator’s performance will degrade with time. Furthermore, the system assumes a subject always walks forward, and is unable to infer rotation and moving direction, which can cause a significant difference between the estimated orientation and actual orientation.

Recently, Deng et al. reported an IMU-aided system to compensate body shadowing error for BLE-based indoor positioning [20]. The effects of the human body on BLE signal RSSI were analysed. A compensation model was proposed which considers the distance and angle between an RN and the unknown target to calculate the error. The distance is calculated from the signal propagation model, and the user’s heading is approximated from the IMU. Finally, an algorithm was proposed to estimate the location of an unknown target by mitigating the body shadowing error in real time. Results demonstrated that the system could achieve an average accuracy of 0.77 m for location estimation. However, the use of IMU exclusively can produce wrong heading estimation because of error accumulation issues with IMU. Moreover, the use of a signal propagation model solely to measure distance can produce high distance error, especially indoors. Thus, the described body shadowing detection strategy can lead to erroneous output as a consequence of the errors from the heading and distance estimation. 

In this study, the user’s orientation is estimated by applying a unique approach using a BSE compensation model that is proposed to mitigate the user’s body shadowing error in real time to improve indoor localisation accuracy.

## 3. Analysis of User’s Body Shadowing Effect on RSSI

Several experiments were performed to investigate the effects of the user’s body on radio signal RSS values. This section describes the experiments and provides observations from the experiments. 

### 3.1. Experiments Overview

**Figure 1:**
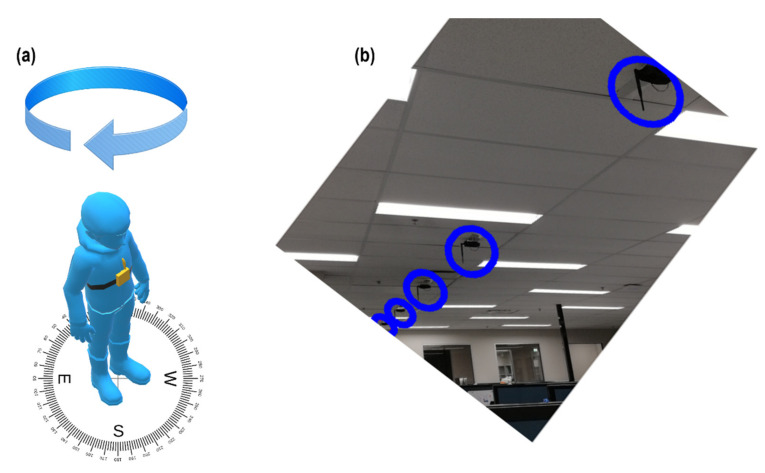
Experimental setup to measure user’s body effect. (**a**) Wearable device attached to the chest of a user; (**b**) reference nodes attached beneath the ceiling (marked by circles).

The experiments were conducted in two different indoor environments with a similar type of setup. The details of the hardware modules used for the experiments are discussed in Section 8.1. Four adults (two males and two females) with different heights, weights, and body shapes participated to allow us to collect data for the experiments. During each experiment, a participant wore a chest-mounted wearable device as shown in Figure 1a. Data were collected from six RNs installed in the ceiling, as shown in Figure 1b, and placed in the same direction 2.1 m, 5.1 m, 7.5 m, 9.9 m, 12.3 m, and 15.6 m away from the participant. There were both the LOS and NLOS communication scenarios between the participants and RNs. A participant collected the RSSI from each RN while standing and turning $360^{{}^{\circ}}$ towards the clockwise direction around the vertical axis. During data collection, a participant turned $15^{{}^{\circ}}$ in every 60 s and collected RSSI data with a frequency of 2 Hz that took 24 min for a complete rotation. The average of the collected RSSI was calculated for every angle position and stored in a database for further analysis. Each participant performed the experiment several times and at different time periods of a day for a month.

### 3.2. Observations

**Figure 2:**
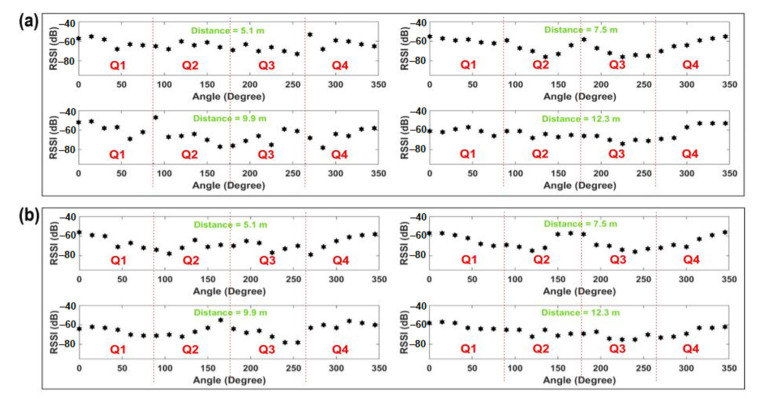
RSSI values versus angle for different distances. (**a**,**b**) illustrate the mean of multiple data points collected by two different participants from two separate indoor environments.

Figure 2 shows the experimental results from two different experiments, performed by a female participant and a male participant in different indoor environments. For each case, the figure presents the average RSSI values for different angles under four specific distances between a participant and the RNs.

As can be observed from Figure 2, there is a similar trend in the ups and downs of the RSSI values for the different angle positions. The general trend shows the highest RSSI values when the body is placed at $0^{{}^{\circ}}$, which is the straight LOS between the transmitter and the receiver. Then, the RSSI values start decreasing with the body’s rotation throughout the first quarter (Q1) and reach the lowest at around the ending of Q1 and the starting of the second quarter (Q2). They then start rising and continue throughout Q2 and reach a small peak at around the ending of Q2 and starting of the third quarter (Q3). Then, again, the RSSI values start falling until the end of Q3 and start of the fourth quarter (Q4) where they reach the lowest once more. After that, the RSSI values continue to increase until they become straight LOS again, where the values reach the peak. Thus, for most of the cases, the lowest RSSI values are found at the angle positions just after the angle $90^{{}^{\circ}}$ and just before the angle $270^{{}^{\circ}}$.

**Figure 3:**
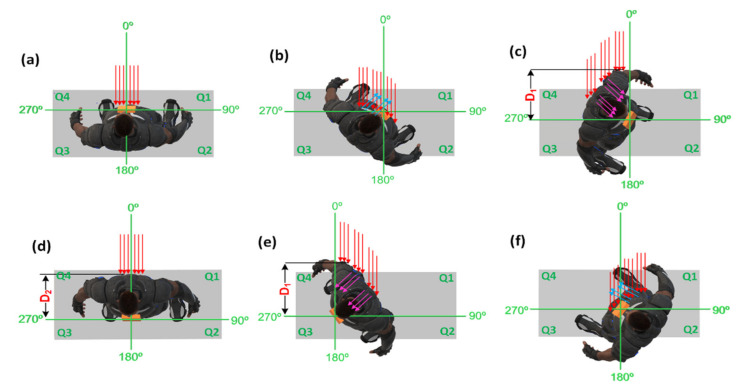
Body position and the status of the electromagnetic waves for different angle positions in each quarter. (**a**) at 0°, (**b**) at 45°, (**c**) just after 90°, (**d**) at 180°, (**e**) just before 270°, and (**f**) at 315°.

To further understand the reason for the results obtained above, Figure 3 illustrates the body position for each quarter, as well as a graphical representation of the electromagnetic waves when arriving at the body and the sensor.

When the body is placed with an angle of $0^{{}^{\circ}}$ with respect to the LOS with the transmitter, as in Figure 3a, the electromagnetic wave arrives directly to the antenna without major interference. The antenna used for the experiments in Figure 1 was a common dipole antenna connected to the XBee receptor node, with vertical orientation. We can assume for $0^{{}^{\circ}}$ that the signal is mostly perpendicular to the chest surface, and therefore also the sensor surface. From 0° to $45^{{}^{\circ}}$ (clockwise orientation), the low variability among the measured RSSIs at the receptor may be explained due to their closeness to the LOS between the transmitter nodes and the body. On the other hand, after the angle values of $45^{{}^{\circ}}$, the variability among the consecutive RSSI values starts to increase, which can be explained using Figure 3b. The body as a transmission medium can be seen as a charged object with higher conductivity (higher loss) and low penetration depth. This low penetration depth means that the signal is highly attenuated inside the conductive body, due to muscles and tissue, and the effect of the electromagnetic wave is highly concentrated on the surface. This influences the signal in such a way that the body guides the surface wave and behaves as a reflector for space waves [22]. These surface waves are explained due to the diffraction of the electromagnetic signal. The diffracted wave’s components are propagated along a curved surface, such as the body [23]. This means, as in Figure 3b, if the signal arrives first to the shoulder, the surface-propagated component can affect the direct vertical electromagnetic components that arrive at the antenna and change the RSSI value. On the other hand, some electromagnetic waves are also reflected on the surface. From all the reflected wave components, the one with the biggest amplitude has the same angle as the incident wave [24]. Such interactions between the reflected, incident, and diffracted waves influence the difference in the values obtained for specific angles and the variability of the data as well. The diffracted and reflected wave components may constructively or negatively interfere with the original signal from the transmitter. This explains the distribution of data observed in Figure 2 for some angles in Q2 and Q3.

In Figure 3c–e, it is shown, for Q1 and Q2, how the shadowing effect affects the value of the RSSI in Figure 2. Depending on the penetration depth and the shape of the human body, the signal is attenuated in a nonuniform way inside the body. This explains, then, the variability and the lower values presented for each distance in Q2 and Q3, especially at the angle positions just after $90^{{}^{\circ}}$ and just before $270^{{}^{\circ}}$. However, something important shall be mentioned. It is observed from Figure 3 that the lowest value on each of the tests is not at $180^{{}^{\circ}}$. As depicted in Figure 3c–e, due to the body geometry and position, the shadowing influence in $180^{{}^{\circ}}$ is physically lower than the shadowing in an angle position in Q2 and Q3. For $180^{{}^{\circ}}$ in Figure 3d, the waveform shall travel through the distance (a length from back to chest) inside the body. In the case of Figure 3c,e, the signals become more attenuated when travelling through a distance D1 (higher than D2, since it is the length from the back part of the shoulder to chest), which explains the results obtained for the values between $195^{{}^{\circ}}$ to $270^{{}^{\circ}}$. Finally, once the body is placed with a heading angle towards Q4 (Figure 3f), the reflected space components and the diffracted surface components can affect (by component cancellation) the RSSI value, with less attenuation than in Figure 3c–e. The last is proved in Figure 2, which shows the trend of increasing in Q4 and the variability of the signal being reduced. Thus, it is clear from the above observations that, on top of the other factors, the user’s body has a significant impact on RF signal and RSS values, which is crucial to consider for RSSI fingerprinting-based indoor positioning applications.

## 4. Overview of the Proposed System

**Figure 4:**
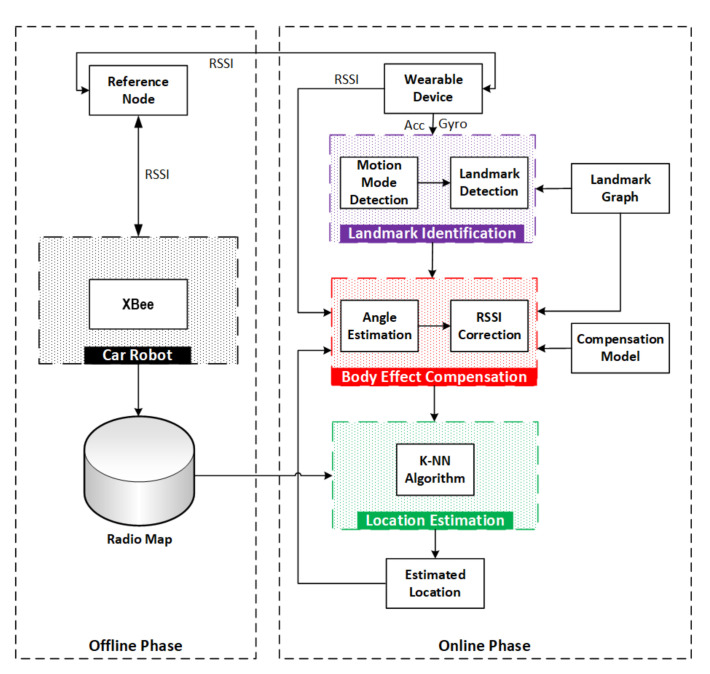
Proposed architecture of RSSI fingerprinting-based indoor localisation with user’s body shadowing compensation.

Figure 4 presents the architecture of the proposed system that follows the traditional fingerprinting scheme. It is mainly composed of two phases: offline training phase and online localisation phase. However, the core contributions lie in the profiling of the query fingerprints by correcting the RSSI and in the fusion of IMU-aided landmarks with classical K-NN method during the online localisation phase. More specifically, when matching a query from a target, the rectification of the queried fingerprint is performed to mitigate the user’s BSE on signal RSSI by leveraging the geometrical features from the indoor floor plan, named landmark.

### 4.1. Offline Training Phase

Although the traditional way of creating a radio map is to partition the area of interest into grids of uniform size followed by the radio fingerprint data collection along a straight path within those grids, this type of manual collection of fingerprint incurs the radio map with RSSIs which have human body shadowing error with them. To exclude this error from the collected RSSI, this study utilised a self-directed car to collect radio fingerprints for radio map. The details of the car, along with its working principle, can be found in our previous work [25]. The car can produce a radio map having nonuniform grids with curved path which have better coverage within the selected area, thus making the system more realistic for real-life localisation applications. Suppose ${\tilde{R}}_{ij}$ is the set of RSS values collected from RN j in the $i^{th}$ entry of the radio map; therefore

(1) $${\tilde{R}}_{ij}=\left\{r_{ijk}:k\in N_{s}\right\}$$

where $r_{ijk}$ is the $k^{th}$ sample and $N_{s}$ is the total number of samples from a particular RN for a specific collection point. Together with the coordinate of the collection point, Equation (1) becomes

(2) $$\left\{x_{i},y_{i},({\tilde{R}}_{ij}:j\in N_{RN}):i\in N_{CP}\right\}$$

where $x_{i}$ and $y_{i}$ are the 2D coordinates of the $i^{th}$ fingerprint, $N_{RN}$ is the total number of RN that can be accessed from $i^{th}$ collection point, and $N_{CP}$ is the total number of individual fingerprint tuples in the radio map. As several RSS measurements are collected from each RN for every RP, the mean value of RSS is calculated as

(3) $$R_{ij}=\frac{1}{N_{s}}\overset{N_{s}}{\underset{k=1}{\sum }}{\tilde{R}}_{ijk}$$

where $R_{ij}$ is the mean RSS value recorded in the database. Therefore, the final formations of an entry in the radio map can be defined as

(4) $$\left\{x_{i},y_{i},\left(R_{ij}:j\in N_{RN}\right):i\in N_{CP}\right\}$$

### 4.2. Online Localisation Phase

As shown in Figure 4, the online phase consists of three main modules: landmark identification module, body effect compensation module, and location estimation module. The landmark identification module detects motion modes from IMU data and recognises indoor landmarks by leveraging a landmark graph. The body effect compensation module estimates the angle between a wearable device and an RN by utilising the detected landmarks, the landmark graph, and the previously estimated location. Then, this module corrects a query fingerprint by compensating the RSSI based on a user’s body shadowing compensation model. Finally, the K-NN algorithm computes the current location of a target by searching the closest match from the radio map based on the corrected query fingerprint. The detailed descriptions of the three modules for the online localisation phase are presented in Section 5, Section 6 and Section 7, respectively.

## 5. Landmark Identification

The basic concept of landmarks and landmark graph are adopted from previous studies [2,6]. Here, landmarks are the sensory markers in the indoor floor plan that encounter specific signal patterns when one or more sensors meet those markers. A landmark can be identified by detecting a subject’s motion modes and by applying a set of rules to those motion modes. Therefore, the landmark identification problem can be described as a motion mode detection problem where the key to efficiently detect a landmark depends on the accuracy of motion mode detection. If the system can detect the motion modes with high accuracy, then the detected motions can be used as inputs to a set of rule-based algorithms to identify the landmarks with high accuracy. Thus, this section mainly focuses on the motion mode detection problem. Before describing the details of the motion mode detection, this section presents an overview of the landmark types along with their detection rules, as well as the landmark graph.

### 5.1. Landmarks and Landmark Graph

Although there are various types of landmarks used for indoor localisation in previous works, this study only considers two types of landmarks: door landmarks and turning landmarks, which are useful for localisation in a 2D indoor environment.

**Figure 5:**
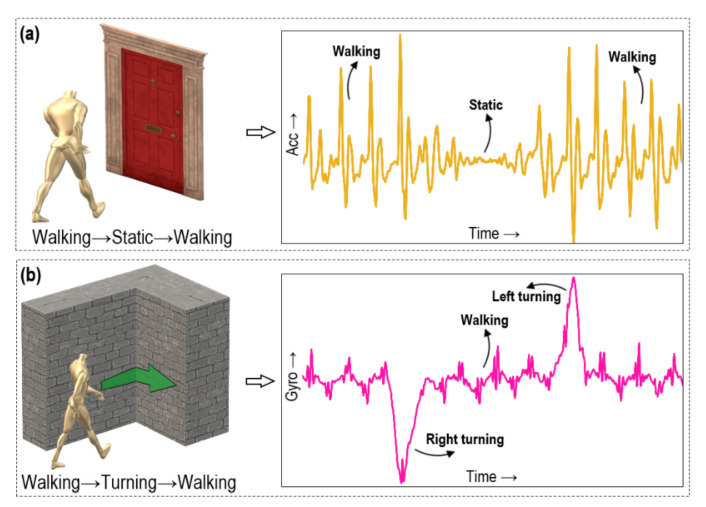
Illustrations and signal patterns of sensory landmarks: (**a**) door landmark; (**b**) turning landmark.

Door landmarks are the sensory markers in the indoor floor plan where the state of motion modes of a subject experience a distinct change in signal patterns. In this work, accelerometer readings are utilised to perceive the door landmarks that can present a specific signal pattern. The usual change pattern in motion modes when accessing a doorway is “walking→static→walking”, which is exploited to infer the door landmark. Figure 5a illustrates the concept of a door landmark that presents a typical change pattern from accelerometer measurement that can be identified by detecting the corresponding motion modes for walking and static activities, and setting a proper time threshold in between the activities. Mathematically, the rule to detect a door landmark, $L_{door}$, can be defined as follows [6]:

(5) $$\begin{array}{l} L_{door}=(\left(x_{t},\;y_{t}\right)\;|\;(mm_{t-Th_{t1}:t}==walk)\;\wedge \;(mm_{t:t+Th_{t2}}\\ \;\;\;\;\;\;\;==static)\;\wedge \;(mm_{t+Th_{t2}:t+Th_{t1}+Th_{t2}}==walk)) \end{array}$$

where $mm_{t}$ is the subject’s motion mode at time t, and $Th_{t1}$ and $Th_{t2}$ are the two time thresholds that regulate the time for the corresponding motion status.

Turning landmarks are the sensory markers in the indoor map where the motion modes provide distinct signal patterns. In this study, data from both the gyroscope and accelerometer combinedly provide a specific motion pattern such as “walking→turning→walking”, corresponding to the direction of turning. Figure 5b shows the idea of turning landmark when a subject takes right and left turns. The rule for detecting a turning landmark can be expressed as follows:

(6) $$\begin{array}{l} L_{turning}=(\left(x_{t},\;y_{t}\right)\;|\;(mm_{t-Th_{t1}:t}==walk)\;\wedge \;(mm_{t:t+Th_{t2}}\\ \;\;\;\;\;\;\;==turn)\;\wedge \;(mm_{t+Th_{t2}:t+Th_{t1}+Th_{t2}}==walk)) \end{array}$$

A landmark graph is a directed graph where the landmarks act as nodes and the path segment between two adjacent landmarks acts as edges. Let $LG=\left(LM,\;TJ\right)$ denote a landmark graph where LM is the set of vertices $\left\{lm_{1},\;lm_{2},\;\ldots lm_{N}\right\}$, i.e., the potential set of landmarks, and TJ is the set of edges $\left\{tj_{1},\;tj_{2},\;\ldots tj_{N}\right\}$, i.e., the potential set of trajectories that connect the landmarks. Each landmark, $lm_{i}$, is represented by its location coordinates, its type, and its unique identifier using the tuple $<x_{i},\;y_{i},\;lt_{i},\;id_{i}>$. Each trajectory $tj_{i}$ is a tuple $<lm_{j},\;lm_{k},\;\theta_{jk},\;d_{i}>$ that connects two adjacent landmarks, with the angle difference relative to the x-axis in anticlockwise direction and the accessible path distance between those landmarks. The locations of these landmarks are acquired from the indoor floor plan, and a landmark graph is constructed.

### 5.2. Motion Mode Detection

#### 5.2.1. Motion Mode Definition

The understanding and detection of motion mode are helpful to implement IMU-assisted indoor localisation. There are different kinds of motions experienced by an IMU which mainly depend on the placement of the sensor on a user’s body. Usually, motion models used by the researchers are specific to the position of IMU installation on the body, e.g., foot-mounted [26], handheld [27], head-mounted [28], etc. In this study, we use chest-mounted IMU for landmark detection; the chance of interference by motions coming from the irregular motion class is minimal because of the steadiness of the device. Moreover, the pose of the device will not vary, as in the case of handheld or head-mounted IMU. Thus, to detect a landmark precisely, the following four types of motion modes were considered in this study.

- Static motion: this type of motion mode includes all the circumstances when a subject is static. A subject will be deemed to be in static mode when his/her spatial position does not change throughout a considered time window. This mode also considers the states as static when a subject obtains slight motion that is not significant enough to infer it as typical locomotion, for example, if a subject moves slightly by stepping on the same spot while opening a door. To detect a landmark correctly, this type of movement must be identified as static.
- Striding motion: this type of motion mode involves the continuous and smooth motion states that contain periodicity and similarity characteristics for a particular time period in their feature set attributes. It includes the motions that change a subject’s spatial position, e.g., plain walking, walking on stairs, or running.
- Turning motion: this refers to the motion states when a subject takes a turn while standing or walking, e.g., performing left or right turning.
- Intermittent motion: this type of motion mode refers to the cases that generate irregular motion states without having the periodicity and continuity properties. It includes all the motion states that a subject performs while remain standing and does not contribute to the change in his/her spatial position, for example, bending or shaking the subject’s body while standing on the same spot.

#### 5.2.2. Motion Mode Classification

**Figure 6:**
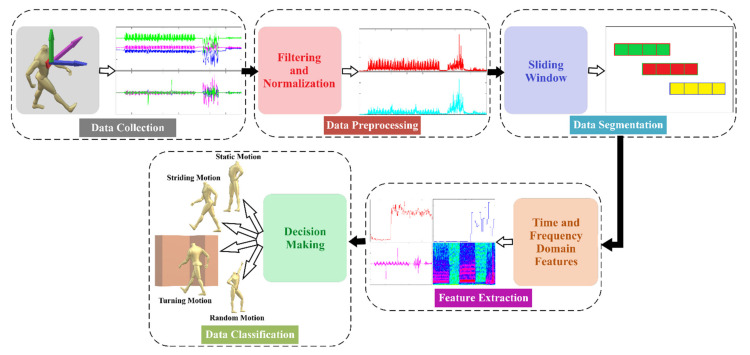
Motion mode detection process pipeline.

The purpose of a typical classification system is to allocate an input pattern automatically to a known set of items based on some decision rules. As shown in Figure 6, usually, a typical classification method is performed using four steps, such as data preprocessing, data segmentation, feature extraction, and decision-making. This study adopted the motion mode classification methods as described in [29]. For data preprocessing, a band-pass filter is used that removes both the unwanted low- and high-frequency noises to focus only on the body movement-contributed signal portion. In this study, a band-pass Butterworth filter of order eight was used to remove both the low- and high-frequency noises. The power spectral analysis of the raw data collected from three-axis accelerometer and three-axis gyroscope was performed to determine the signal and noise characteristics. As analysed, most of the energy related to human motion captured by the accelerometer and gyroscope was between 0.75 Hz and 25 Hz. Thus, these two frequencies were applied as the cut-off frequencies to the filter for removing the low- and high-frequency noises, respectively. In this study, though the sensor is body fixed, it can slightly change its orientation regarding the original setup in the x-, y-, and z-axis, because of the generated motions during experiments. This change in sensor orientation affects the acceleration in various degrees towards the x, y, and z coordinate systems by decomposing the gravitational component [30]. To eliminate the dependency on device orientation, the normalised magnitude of the IMU data was considered in this study. As the human movement is a continuous process over time, an individual data point cannot reflect a complete motion mode of a user. Thus, to extract features that can characterise a motion mode, collected sensor data need to be segmented into sequences within a certain time frame, named a window. Here, a window size of 2 s with 50% overlapping was selected, which translates 200 samples for a sampling frequency of 100 Hz. The choice of this type of window is typical and often utilised for motion mode detection, which has already been validated by previous studies [29,31,32]. As this study only considers four types of motion modes, the feature set proposed by Susi et al. [29] was used, which can manage the trade-off between the classification performance and computation cost. The features were extracted from the preprocessed windowed data, which include the energy of the accelerometer and gyroscope, the variance of the accelerometer and gyroscope, and the dominant frequencies of the accelerometer and gyroscope. In addition to those features, this work considered the change of angular velocity along the vertical axis to detect the turning motion.

**Figure 7:**
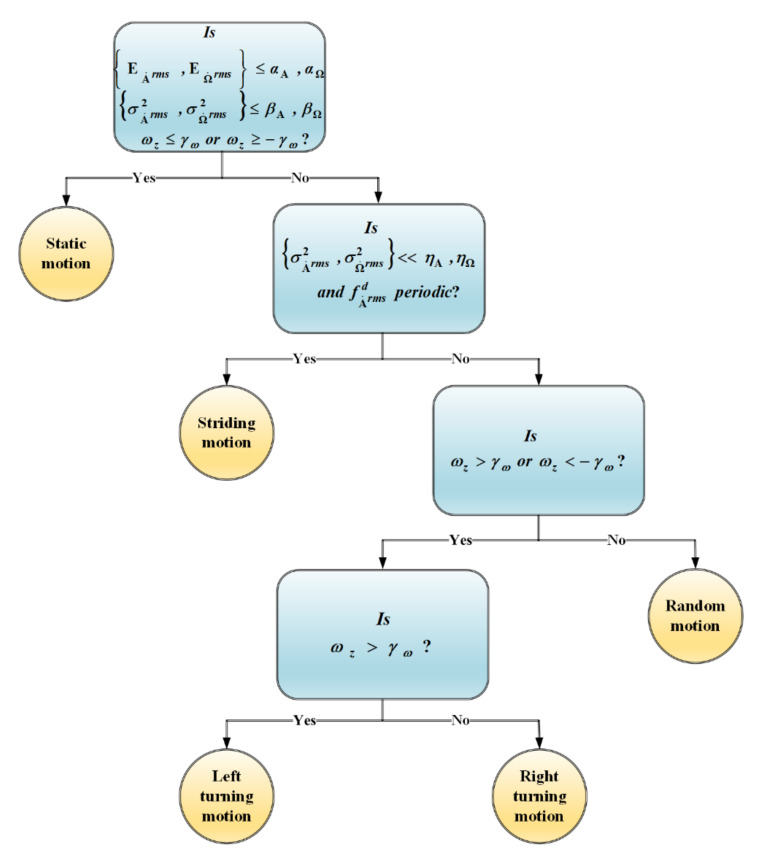
Structure of the decision tree to classify various motion modes.

As proposed by [29], this work utilised a supervised approach of classification named decision tree for performing the classification task. A decision tree forms a tree-like mapping that comprises leaf nodes, representing the classes, and internal nodes that symbolise the tests regarding the features. The internal nodes contain one (i.e., univariate) or multiple (i.e., multivariate) conditional control statements, and traversing the tree from the root node to leaf nodes can classify a given input pattern. Figure 7 presents the decision tree that was used in this study to detect the abovementioned motion modes. The threshold values for each feature set in every internal node is set by the classifier after performing the training. Initially, the tree characterises the static and dynamic types of motion modes based on the energies and variances of the accelerometer and gyroscope, as well as the raw angular velocity along the vertical axis. As the signal variances for the random movement are significantly higher in short temporal periods than the striding motion, it is utilised to separate the striding motion from other dynamic motions. Moreover, to ascertain whether that motion is from a periodic activity, the periodicity of the dominant frequencies of the accelerometer is evaluated, which reflects the periodic motions generated from human gait. However, the striding motion can be further divided into other classes, such as plain walking, fast walking, running, walking up or down stairs, etc., for other aims that are beyond the scope of this study. Finally, the turning motions are differentiated from other random motions by evaluating the angular velocity of the raw data along the vertical axis.

## 6. User’s Body Shadowing Effect Compensation

There are two main parts to compensate the effect of user’s body shadowing on RSSI calculation: angle estimation and compensation model.

### 6.1. Angle Estimation

**Figure 8:**
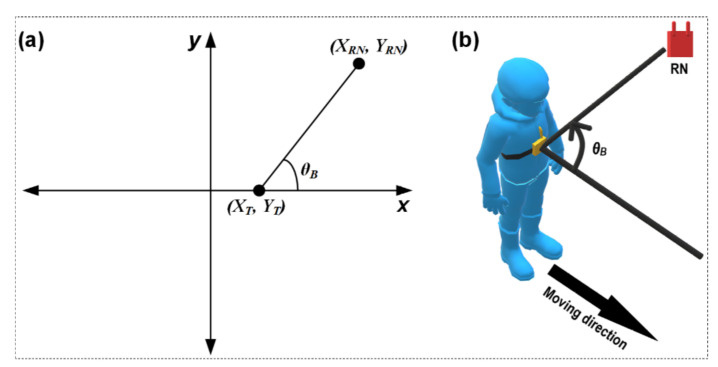
Angle estimation between a wearable device and an RN: (**a**) concept of atan2; (**b**) adaptation of atan2 for estimating the angle between the moving direction and the RN.

Because of the geometrical structures of the indoor environment, the movement of people indoors usually tends to be in the same direction at least for a few seconds, e.g., in the case of a corridor, a user can walk in two directions. As the usual direction of a chest-mounted wearable device is the same as the movement direction of a user, this behaviour, along with the concept of landmark graph, RNs location information, and indoor environmental geometrical constrain, can be exploited to calculate the angle between the chest-mounted wearable tag and the RNs. To calculate the angle between a user and an RN, the two-argument arctangent function (atan2) is used, which can estimate the angle in the Euclidean plane between the positive x-axis and a line connecting to a point, as shown in Figure 8a.

In this study, the path segment where the user is walking during the angle estimation is considered as the line corresponding to the x-axis. Thus, the angle is calculated between the moving path and the line connecting the current location of the user and the location of the RN (Figure 8b), as follows:

(7) $$\theta_{rad}=atan2\left(Y_{RN}-Y_{T},\;X_{RN}-X_{T}\right)\in \left(-\pi ,\;\pi \right)\;\mathrm{and}\;\left(X_{RN},\;Y_{RN}\right)\neq \left(0,0\right)$$

(8) $$\theta_{deg}=\frac{180^{{}^{\circ}}}{\pi }\;\theta_{rad}$$

(9) $$\theta_{B}=\left\{\begin{array}{cc} \theta_{deg}, & \mathrm{when}\;\theta_{deg}\geq 0\\ 2\pi +\theta_{deg}, & \mathrm{when}\;\theta_{deg}<0 \end{array}\right.$$

where $\left(X_{T},\;Y_{T}\right)$ and $\left(X_{RN},\;Y_{RN}\right)$ are the coordinates of the current locations of the user and the RN, and $\theta_{B}$ is the bearing angle between the user and an RN in degrees. In this study, a coarse location of the unknown target is estimated first as the current location $\left(X_{T},\;Y_{T}\right)$ to estimate the bearing angle between the target and an RN. This coarse location is computed based on its immediate previous location, and the step length and moving direction. Here, the step length and step direction are obtained by leveraging the landmark graph. At the start, the step length for a target is initialised to a constant value. As the target progresses and passes two adjacent landmarks, the step length is updated. Let a target pass two adjacent landmarks denoted by $L_{1}$ and $L_{2}$. Then, the step length of that target can be obtained as follows:

(10) $$l_{s}=\frac{\sqrt{{\left(x_{L_{1}}-x_{L_{2}}\right)}^{2}+{\left(y_{L_{1}}-y_{L_{2}}\right)}^{2}}}{N_{S}}$$

where $\left(x_{L_{1}},\;y_{L_{1}}\right)$ and $\left(x_{L_{2}},\;y_{L_{2}}\right)$ are the coordinates of the landmarks $L_{1}$ and $L_{2}$, respectively, and $N_{S}$ is the total number of previous location points in between the landmarks $L_{1}$ and $L_{2}$ that are estimated when the target passes the landmarks. Here, the step length will only be updated if the trajectory between the adjacent landmarks is a straight line. Otherwise, the system will retain the step length that estimated last. The estimation of step direction exploits the geometrical structures of indoor environment and infers the moving direction as the direction of the current trajectory relative to the considered x-axis. Thus, when the step length $l_{s}$ and heading $\theta_{s}$ are known, the estimation of the next location can be obtained by using the elementary pedestrian dead reckoning (PDR) technique as follows:

(11) $$x_{s+1}=x_{s}+l_{s}\;sin(\theta_{s})$$

(12) $$y_{s+1}=y_{s}+l_{s}\;\cos\left(\theta_{s}\right)$$

where $\left(x_{s},\;y_{s}\right)$ and $\left(x_{s+1},\;y_{s+1}\right)$ are the positions of a subject at step s and s+1, respectively, and $\theta_{s}$ and $l_{s}$ are the heading and displacement at step s. Therefore, $\left(x_{s+1},\;y_{s+1}\right)$ are considered as the current location $\left(X_{T},\;Y_{T}\right)$ to calculate the angle between an RN and the current coarse position of the target, considering the target is moving exactly the same direction as the trajectory’s direction. However, a target can rotate his/her body while walking towards a trajectory’s direction, which will eventually affect the measured $\theta_{B}$ in the perspective of RSSI correction. Let $\theta_{R}$ be the rotation angle obtained from the gyroscope. Then, the orientation angle $\theta_{O}$ of the target relative to an RN can be calculated as follows:

(13) $$\theta_{O}=\theta_{B}\pm \theta_{R}\pm \theta_{L_{1}L_{2}}$$

where $\theta_{L_{1}L_{2}}$ is the angle difference of the path segment connecting the landmarks $L_{1}$ and $L_{2}$ relative to the x-axis in anticlockwise direction, which can be obtained from the landmark graph.

### 6.2. Compensation Model

To compensate the effects of the user’s body on signal RSSI value, a compensation model is proposed in this study. This model can intensify the signal RSSI values that are being interrupted by the user’s body. Thus, the proposed model for correcting the raw RSSI is as follows, which considers the angle between the user’s body and the RN:

(14) $$RSSI_{corr}=RSSI\left(-\frac{\sigma }{d}e^{\frac{-{\left(\theta_{O}\right)}^{2}}{2\theta_{O}^{2}+1}}+1\right)$$

where RSSI and $RSSI_{corr}$. are the raw and corrected RSSI values, $\theta_{O}$ is the orientation angle between the user and an RN in degrees, σ is the intensification parameter, and d is the distance between the wearable tag and the edge of the far-ended shoulder. The value of σ depends both on the environment and the user orientation with respect to the RN, which needs to be chosen empirically.

However, as observed from Figure 3, the corrected RSSI will be an overestimation when applying the proposed compensation model with the straight LOS direction (e.g., around $0^{{}^{\circ}}$) between the wearable tag and the RN. Moreover, it will be an underestimation when the signals are being interrupted by the maximum obstacles (e.g., the angle positions just after the angles $90^{{}^{\circ}}$ and just before the angle $270^{{}^{\circ}}$). To resolve this issue, the following rules with different values of the parameter σ are chosen for different angles:

(15) $$\sigma =\left\{\begin{array}{ll} \sigma_{1}, & if\;(0^{{}^{\circ}}\leq \theta_{B}\leq 45^{{}^{\circ}})\;||\;(315^{{}^{\circ}}\leq \theta_{B}\leq 360^{{}^{\circ}}),\\ \sigma_{2}, & if\;(45^{{}^{\circ}}<\theta_{B}\leq 135^{{}^{\circ}})\;||\;(225^{{}^{\circ}}<\theta_{B}\leq 315^{{}^{\circ}}),\;\\ \sigma_{3}, & if\;(135^{{}^{\circ}}<\theta_{B}\leq 225^{{}^{\circ}}). \end{array}\right.$$

To estimate the values of σ, this study first calculates the amount of error ($e_{\theta }$) as given in Equation (16), for a given range of angles by choosing a value of σ. Secondly, the optimum value of σ is estimated by adjusting its value untill the smallest $e_{\theta }$ is obtained.

(16) $$e_{\theta }=\frac{\sum_{i=0}^{n}\;\sqrt{{\left(RSSI_{\theta_{s}+i*m}-RSSI_{0^{{}^{\circ}}}\right)}^{2}}}{\theta_{e}-\theta_{s}}\times 100\%$$

where $RSSI_{\theta_{s}+i*m}$ and $RSSI_{0^{{}^{\circ}}}$ are the RSSI values at angle $\theta_{s}+i\times m$ and $0^{{}^{\circ}}$, respectively, n is the total number of angle values considered to collect data within the range $\theta_{s}$ to $\theta_{e}$, and m is the amount of angle considered to rotate in each move. As this study considers collecting RSSI data at every $15^{{}^{\circ}}$ rotation, the value of m is 15.

## 7. Location Estimation

Irrespective of the utilised features, a fingerprinting-based localisation problem is mainly a pattern matching problem. During the online phase, a target sends a query fingerprint from an unknown location that needs to be matched with the fingerprints stored in the radio map. It is very unlikely that a radio map will contain a fingerprint with an exact match. Thus, the traditional way is to find K different fingerprints closest to the queried one from the radio map, which is known as the K-nearest neighbour (K-NN) method. This study exploits the classical K-NN algorithm for location estimation. Moreover, an improved version of the classical K-NN algorithm, named weighted K-NN (WK-NN), is applied to further improve the localisation accuracy. 

Let $r_{uj}$ be the body shadowing-compensated mean RSSI collected from RN j at unknown target location u. Then, the RSSI distance between an RP i from the radio map to the target point u can be obtained as follows:

(17) $$d_{ui}=\frac{1}{N_{RN}}\sqrt{\overset{N_{RN}}{\underset{j=1}{\sum }}{\left(r_{uj}-r_{ij}\right)}^{2}}$$

where $d_{ui}$ is the Euclidean distance between the RSSI of the target point and RP, $r_{ij}$ is the mean RSSI collected from RN j at RP i, and $N_{RN}$ is the total number of RNs considered for a fingerprint. In the case of the K-NN method, K RPs from the radio map will be selected that have the smallest distance value with the target. Therefore, the estimated location of the target can be calculated as follows:

(18) $$LOC\left(x_{u},\;y_{u}\right)=\frac{1}{K}\overset{K}{\underset{i=1}{\sum }}LOC\left(x_{i},\;y_{i}\right)$$

where $\left(x_{i},\;y_{i}\right)$ are the locations of the RPs. Here, the spatial distances between a target location and its neighbouring RPs are usually different. Thus, the WK-NN algorithm also considers the corresponding spatial distances in terms of weight factor when selecting the K nearest RPs. As proposed in [33], the weight is inversely proportional to the spatial distance and can be calculated as follows:

(19) $$w_{ci}=\frac{1/D_{ci}}{\sum_{i=1}^{K}1/D_{ci}}$$

where $D_{ci}=\sqrt{{\left(x_{c}-x_{i}\right)}^{2}+{\left(y_{c}-y_{i}\right)}^{2}}$ is the spatial Euclidean distance between the coarse location $\left(x_{c},\;y_{c}\right)$ of the unknown target and an RP location $\left(x_{i},\;y_{i}\right)$. Therefore, the estimated location will be

(20) $$LOC\left(x_{u},\;y_{u}\right)=\frac{\sum_{i=1}^{K}w_{ci}LOC\left(x_{i},\;y_{i}\right)}{\sum_{i=1}^{K}w_{ci}}$$

In this study, the coarse location of an unknown target is estimated first to calculate the spatial distance between the RPs and a target location. The coarse location of the target is computed by leveraging the landmark graph and using the same technique proposed in Section 6.1. Therefore, the estimated coarse location is used to calculate the weight for the WK-NN algorithm.

## 8. Experimental Evaluation

This section presents the evaluation of the proposed models through quantitative experimental results.

### 8.1. Experimental Setup

**Figure 9:**
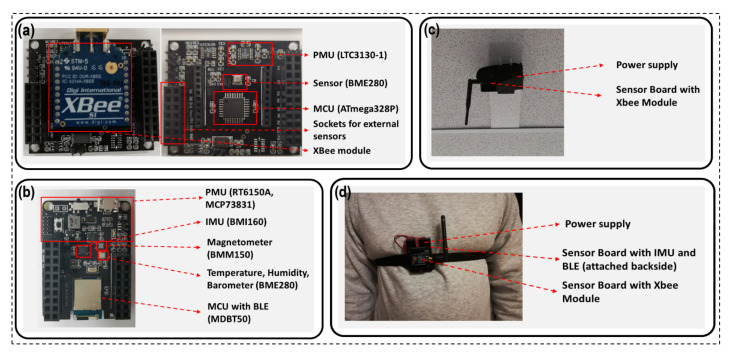
Experimental setup: (**a**) sensor board with XBee wireless module; (**b**) sensor board with IMU and BLE modules; (**c**) RN mounted beneath the ceiling; (**d**) wearable device mounted on chest.

The experiments were carried out using two customised sensor boards designed by our group. To collect the RSSI fingerprint, the XBee wireless technology-based XBee S1 802.15.4 module was used as the radio in the first sensor board, as shown in Figure 9a, which was employed both in the RNs and wearable devices. To acquire the acceleration and angular velocity data, the second sensor board was used as part of the wearable node. This sensor board is equipped with IMU and BLE, as well as some environmental sensors, as shown in Figure 9b. The RNs were attached to the ceiling, as shown in Figure 9c. The wearable sensor boards were attached to a subject’s chest and fastened by an elastic strap, as presented in Figure 9d. A computer connected with an XBee module acted as a server that collected and stored both the RSSI fingerprint data through XBee and IMU data through BLE. In the case of the indoor localisation, the data collected from both the devices were synchronised by using the timestamps and stored on a database for further processing.

### 8.2. Evaluation of Motion Mode Detection

#### 8.2.1. Data Collection

Several experiments were carried out for collecting an adequate amount of data with ground truth labels to train and test the designed motion mode classifier. Data from four adults (two males and two females) of different heights and weights were collected to evaluate the performance of the proposed classifier. The data collections were conducted in an open field, and each participant was equipped with a chest-mounted IMU device. The participants were requested to walk approximately 500 m of distance while performing several activities. The experiments were performed using a predefined protocol that consisted of six activities including static, walking, turning right, turning left, opening a door, bending, and random movement. As the data were collected from an open field, the participants were asked to simulate the door-opening activity. To complete one run of the defined protocol, a participant required four minutes, and each participant repeated the protocol three times. Thus, enough data was collected for training and testing of the classifier. 

To facilitate the labelling of ground truth, another IMU device, named NGIMU (https://x-io.co.uk/ngimu/ (accessed on 10 February 2021), was utilised. Pressing the power button of the device while powered on will send a message to the receiver with timestamped information for the button-pressing event. During the experiment, the participants were asked to press the button of the NGIMU each time they began an activity. Thus, by annotating the exact number and sequence of performed activities and mapping this information with the timestamped button event’s data, the ground truth labelling was performed. For precise evaluation of the classifier, the data recorded during the transition of two activities were removed manually.

#### 8.2.2. Evaluation Metrics

To validate the skill of the proposed classification model, five-fold cross-validation was applied, which is a typical resampling technique that shuffles the dataset randomly and splits it into five equal-sized groups. From those, four groups were used for training the model, and one group was kept for testing the model. Five iterations were performed with the grouped data to cross-validate the model by using each group as a testing dataset while employing the others for training. The average of the evaluation metrics from the five iterations was taken as the final evaluation score of the model. To evaluate the motion mode classification performance, several metrics were used in this study, including accuracy, precision, sensitivity, specificity, and F-measure. The accuracy for a motion class is the ratio of correctly labelled motion modes for that class to the total number of labelled motion modes for that class. The precision for a motion class can be defined as the ratio of correctly positive-labelled motion modes for that class to the total number of positive-labelled motion modes for that class. The sensitivity (which is also known as recall) for a motion class is the ratio of correctly positive-labelled motion modes for that class to the total number of motion modes that actually belong to that class. The specificity for a motion class is the ratio of correctly negative-labelled motion modes for that class to the total number of motion modes that do not belong to that class. F-measure is the harmonic average of precision and sensitivity. These metrics are defined as follows:

(21) $$Accuracy=\frac{TP+TN}{TP+FP+FN+TN}$$

(22) $$Precision=\frac{TP}{TP+FP}$$

(23) $$Sensitivity=\frac{TP}{TP+FN}$$

(24) $$Specificity=\frac{TN}{TN+FP}$$

(25) $$F-measure=2*(\frac{Precision*Sensitivity}{Precision+Sensitivity})$$

#### 8.2.3. Classification Performance

**Figure 10:**
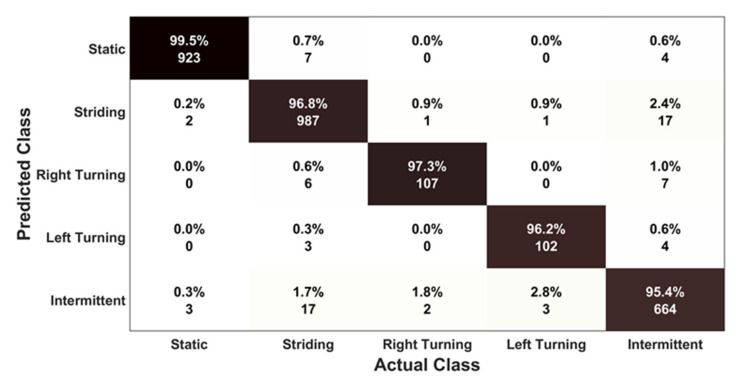
Confusion matrix of the proposed motion mode classifier.

**Figure 11:**
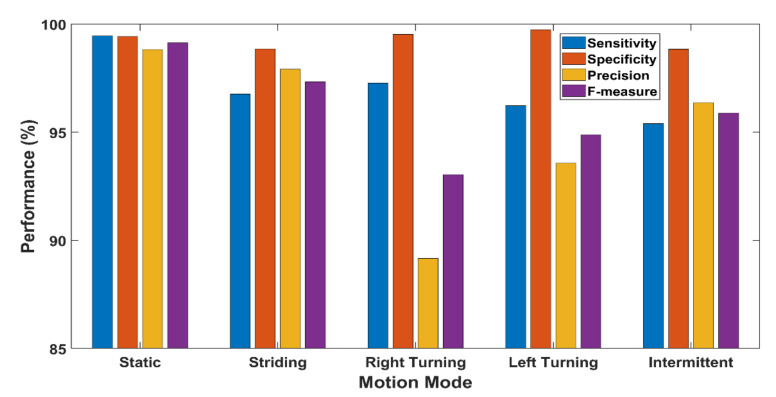
Classification performance of the proposed motion mode classifier for each motion mode.

Figure 10 and Figure 11 summarise the classification performance of the proposed motion mode classifier for detecting each motion mode. As illustrated in Figure 10, the columns of the confusion matrix refer to the ground truth motion modes performed by the participants, and the rows refer to the motion modes predicted by the classifier. The percentage of prediction accuracy, together with their actual number for each motion mode, is presented along the principal diagonal in black colour. The percentage of confused classification for the motion modes are reported along the off-diagonal sections in white colour.

As can be seen from the confusion matrix, the classifier can detect the correct motion mode in more than 95% of cases, irrespective of the type of motion class performed by a participant. The highest accuracy of 99.5% was attained by the classifier for the static motion mode. As reported, 2 and 3 segments out of 928 segments for static type motions were misclassified as striding motion and intermittent motion, respectively. The main reason behind this confusion is because of the simulated door opening activities performed by the participants, which are actually considered as static activities; however, this type of activity sometimes may generate high energy and variance in the signal which can satisfy the decision thresholds, leading to misclassification.

The lowest accuracy of 95.4% was achieved for detecting the intermittent motion mode by the classifier. This is because intermittent motions are more likely to be confused as other types of motions. As reported in the confusion matrix, among the total 696 segments for intermittent motions, 17 were detected as striding motion. One possible reason for this confusion may be the consecutive occurrence of a similar type of movement several times (e.g., multiple bending activities), which can produce periodicity and lead to that misclassification. However, during the landmark identification phase, it is very unlikely that such intermittent movement will occur in a pattern that can satisfy the landmark rules. Thus, the defined rules for landmark identification can mitigate the impact of this type of misclassification. Moreover, 17 segments out of 1020 for the striding motion mode were misclassified as intermittent motion, causing the accuracy for that class to be 96.8%.

As presented in Figure 11, the overall sensitivity, specificity, precision, and F-measure of the proposed motion mode classifiers are 97.03%, 99.28%, 95.17%, and 96.06%, respectively, which can eventually produce high accuracy for the landmark identification task.

### 8.3. Evaluation of User’s Body Shadowing Effect Compensation Model

**Figure 12:**
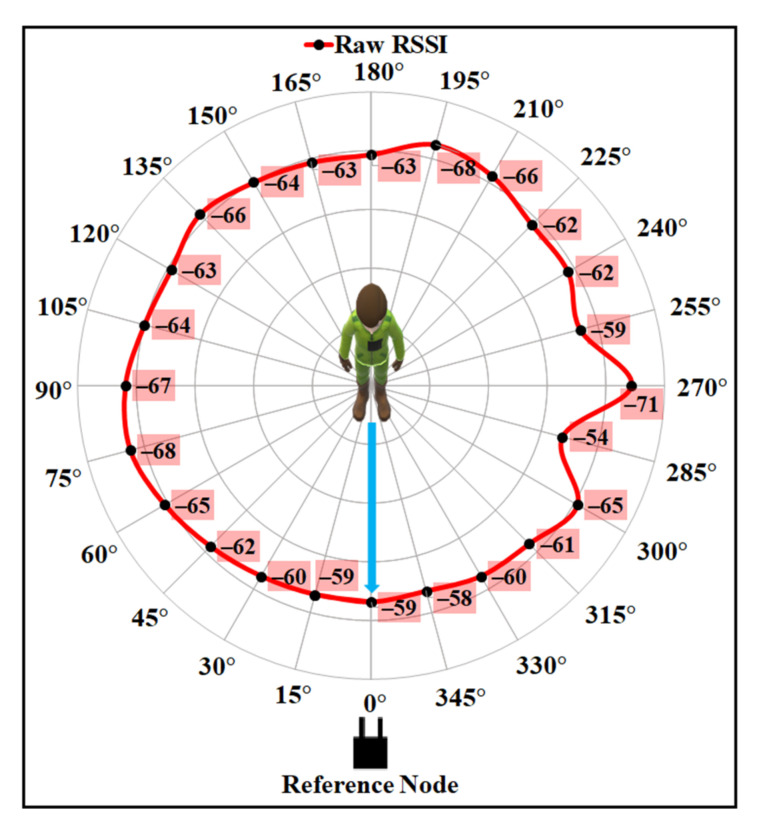
User’s body effect on RSSI (raw RSSI vs. orientation angle).

The proposed BSE compensation model was evaluated firstly using the same experimental data as shown in Figure 2. The polar plot for the raw data is shown in Figure 12, where the distance between the sender and the receiver is 9.9 m. It is noticeable from the figure that the minimum and maximum attenuation of RF signals occur at the angle positions as discussed in Section 3.2, due to the effect of the user’s body. The higher RSSI value is observed at approximately $0^{{}^{\circ}}$ with LOS angle positions, and the lowest values of RSSI can be observed at approximately $90^{{}^{\circ}}$ and $270^{{}^{\circ}}$ angle positions for the raw RSSI data.

**Figure 13:**
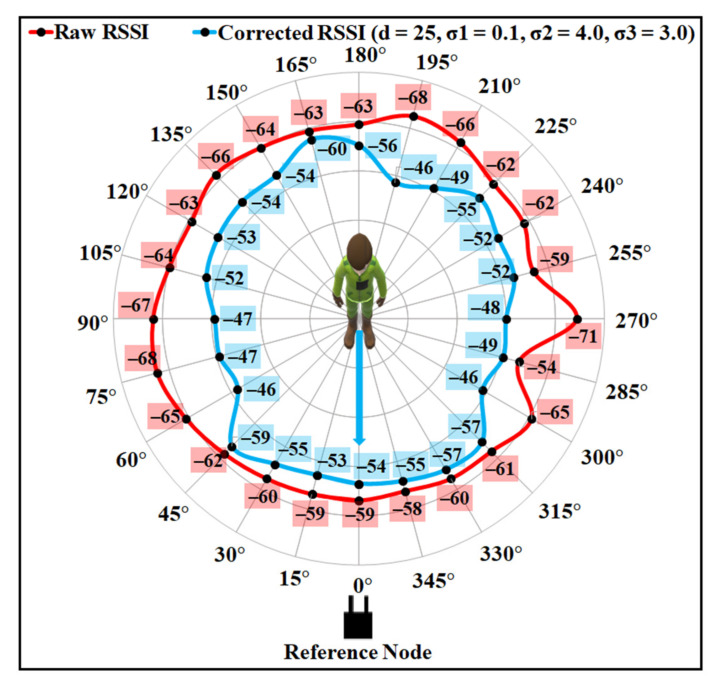
Results after applying the body shadowing effect compensation model with $\sigma_{1}=0.1,\;\sigma_{2}=4.0,\;\sigma_{3}=3.0,$ and d=25.

**Figure 14:**
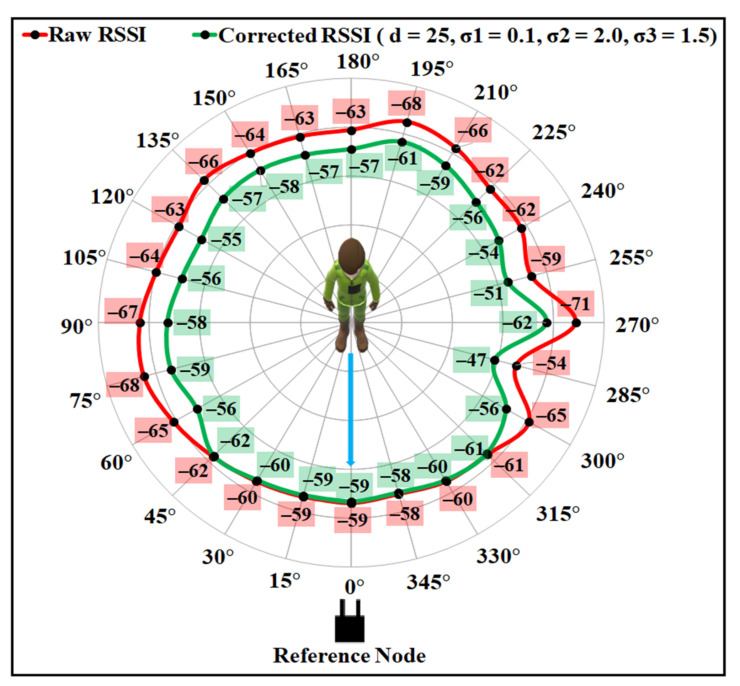
Results after applying the body shadowing effect compensation model with $\sigma_{1}=0.1,\;\sigma_{2}=2.0,\;\sigma_{3}=1.5,$ and d=25.

As the human body shape is uneven, the amount of RSSI depletion, while facing the different body parts, by the signal will be different. The intensification parameter σ boosts the RSSI with an amount that can cope with the loss caused by the user’s body. Using a single value of σ for every orientation angle may lead to an overestimation or an underestimation for some orientations. Thus, three different σ values were chosen based on the analysis, as described in Section 3. To evaluate the compensation model performance, firstly the values for the intensification parameters σ were empirically investigated, and optimal values were selected, as described in Section 6.2. Figure 13 and Figure 14 present the results after applying the proposed compensation model for two different combinations of values for σ. To show the effect of σ on the proposed compensation model, first the values of $\sigma_{1}$, $\sigma_{2}$, and $\sigma_{3}$ were chosen as 0.1, 4.0, and 3.0, respectively. Figure 13 shows the polar plot for the compensated RSSI after applying those values to corresponding orientation angles. As can be noticed from the figure, though the model can correct some RSSI values by applying this set of σ values that are affected by the user’s body shadowing, the corrected values are the underestimation of the $0^{{}^{\circ}}$ faced LOS value. Figure 14 presents the results with the values of $\sigma_{1}$, $\sigma_{2}$, and $\sigma_{3}$ as 0.1, 2.0, and 1.5, respectively, which were found as the optimal values for the experimented indoor environments. As can be seen from that figure, the proposed model can considerably correct the attenuated RSSI values while presenting a negligible amount of noise. Most of the corrected RSSI values are almost similar to the $0^{{}^{\circ}}$ faced LOS value with a negligible deviation. Thus, the values of $\sigma_{1}$, $\sigma_{2}$, and $\sigma_{3}$ were chosen as 0.1, 2.0, and 1.5 to compensate the user’s body affected RSSI values for the proposed indoor localisation system.

**Figure 15:**
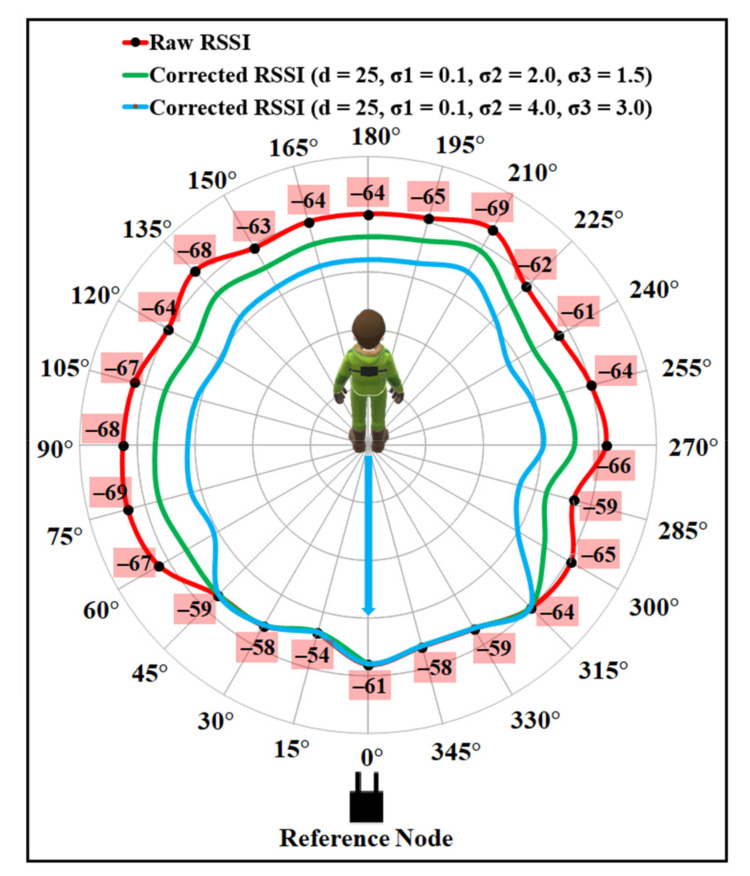
Raw and corrected RSSI for data collected with back-mounted wearable device.

To validate the efficiency of the proposed model, RSSI data were also collected using the same experimental setup, except the wearable device was placed on the back of a user. Figure 15 illustrates the compensation results along with the raw RSSI for the two different sets of σ values. As can be seen from the figure, the values of $\sigma_{1}$, $\sigma_{2}$, and $\sigma_{3}$ as 0.1, 2.0, and 1.5 can produce the best estimates for most of the angle positions, compared to the other set.

**Figure 16:**
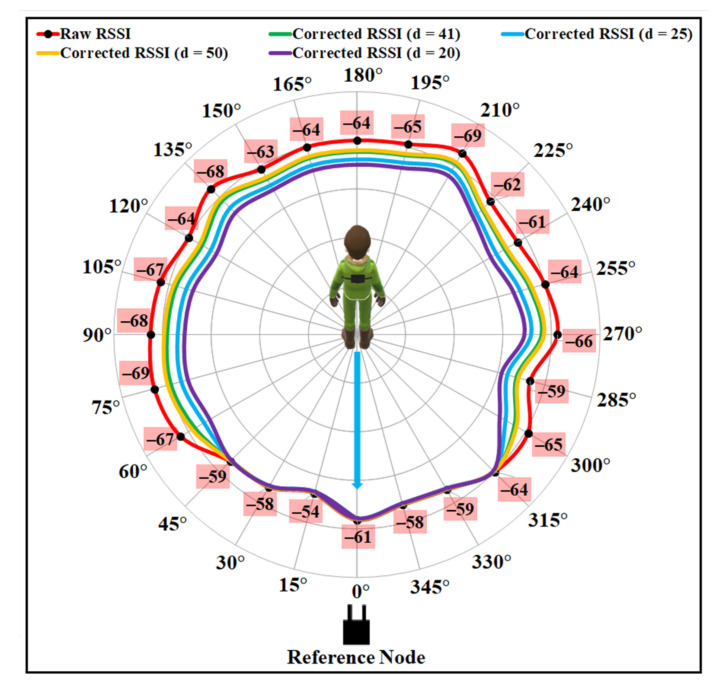
Results after applying different values of d with $\sigma_{1}=0.1,\;\sigma_{2}=2.0,\;\sigma_{3}=1.5$.

The impacts of the parameter d, which represents the distance between the wearable tag and the edge of the far-ended shoulder, were also examined. Figure 16 presents the results of the proposed model for different d values, including 41, 25, 50, and 20. Considering the average shoulder width of 41 cm, selecting the value of d as 25 produces the best results demonstrated in the figure.

### 8.4. Evaluation of the Proposed Localisation System

**Figure 17:**
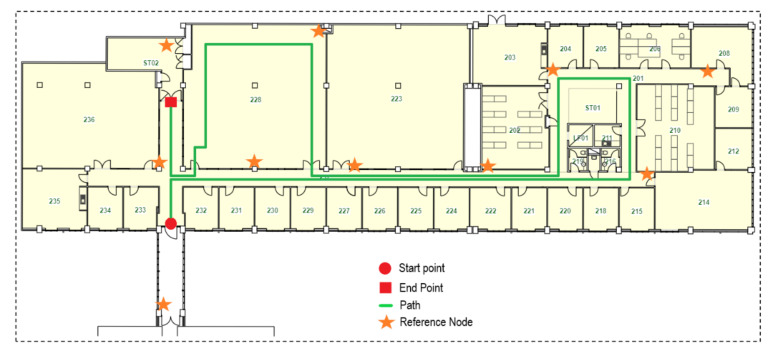
Experimental area with the markings of experimented path and distribution of reference nodes.

To evaluate the performance of the proposed localisation system, experiments were performed in an office building. The experiment building was Building 72 at the Clayton campus of Monash University, which comprises three floors. The experiment was carried out on the second floor, with a total area of about 1475 $m^{2}$, and the length of the test path was about 150 m, represented by the green line in Figure 17. The path starts from the red circle, then follows the path, and ends at the red square.

#### 8.4.1. Evaluation Criterion

To evaluate the system performance, 40 key points were set along the experimented path, which served as the ground truth for comparison. During the experiment, the participant was asked to stop a little at each key point for the purpose of recording the markers. Let $\left(x_{e}^{i},\;y_{e}^{i}\right)$ and $\left(x_{g}^{i},\;y_{g}^{i}\right)$ be the estimated location of an unknown target, and the ground truth location at the ith key point, respectively; then the error at ith key point is

(26) $$\varepsilon_{ge}^{i}=\sqrt{{\left(x_{g}^{i}-x_{e}^{i}\right)}^{2}-{\left(y_{g}^{i}-y_{e}^{i}\right)}^{2}}$$

This study reports the mean error, median error, standard deviation, 25th, 75th, and 90th percentile errors, and a cumulative distribution function (CDF) plot to analyse the localisation performance of the proposed system.

#### 8.4.2. Localisation Performance

The localisation performance of the proposed system was compared with two other studies that proposed different models for angle estimation and BSE compensation, including the work presented in [17], referred to as MODEL I, and in [20], referred to as MODEL II. To compare the relative accuracy of the proposed system with these studies, we mainly implemented the angle estimation and compensation models as proposed by the researchers, and then applied the fingerprinting localisation method. For example, the use of multiple wearable devices and Semcad simulation parts were omitted for MODEL I, and Kalman filter and path loss model implementation were overlooked for MODEL II. Moreover, identical preprocessing of the raw data was considered, and the same weight calculation method was applied for all the cases with a value of 3 for k, both in the K-NN and WK-NN algorithms.

**Figure 18:**
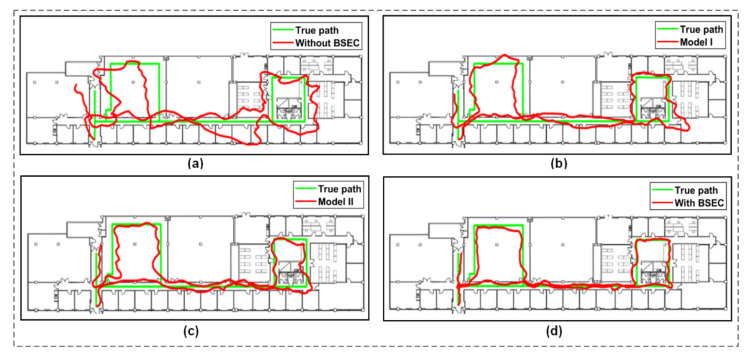
Routes estimated by different systems using the K-NN method: (**a**) Without BSEC; (**b**) Model I; (**c**) Model II and (**d**) With BSEC.

Figure 18 presents the routes for the experimented path estimated by the different systems using the classical K-NN algorithm. As shown in the figure, the performance of the systems with BSE compensation (WBSEC) on indoor localisation is apparent and the estimated route of the systems without BSE compensation (WOBSEC) produced the worst path. Among the other results, MODEL II generated a better path compared to MODEL I, because in MODEL I the authors only considered two groups for orientation angle (over/underestimation) when applying their compensation model. MODEL II considered three groups (front, back, side) and there was no consideration of the volume of body parts that creates the NLOS scenarios. However, this work considered three groups as well as the consideration of body volume when calculating the compensation value, and hence produced the best path.

**Figure 19:**
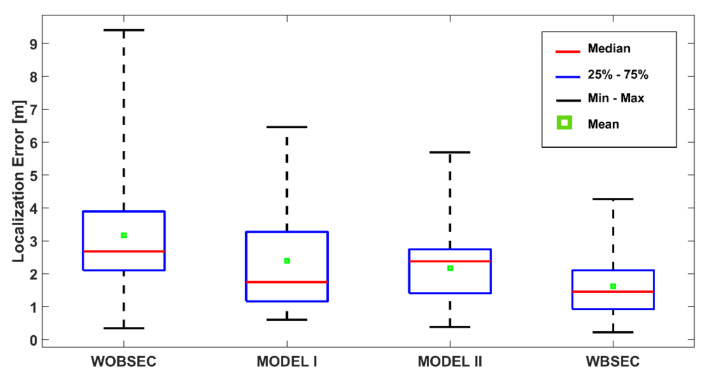
Localisation performance of the proposed system compared with other systems using the K-NN method.

The details of the positioning performance are presented in Figure 19 and Table 2 for the K-NN algorithm. The boxplot indicates the summary of some error statistics including the maximum, minimum, 25th, 75th, mean, and median errors for each system. As presented in Figure 19 and Table 2, the proposed system significantly outperformed the other systems for all types of statistics. The proposed system demonstrated the best performance with a mean error of 1.62 m and median error of 1.46 m, followed by MODEL II (mean error 2.17 m and median error 2.38 m). The mean and median errors of MODEL I were 2.39 m and 1.75 m, respectively. The system without any BSE compensation achieved a mean error of 3.17 m and a median error of 2.68 m.

**Figure 20:**
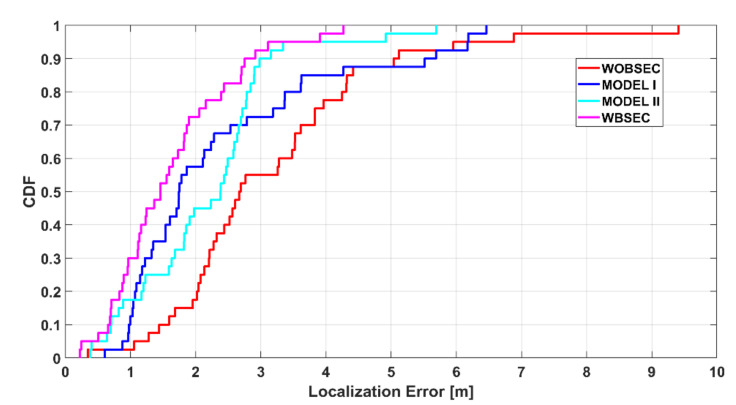
Accumulative distribution of positioning error of the proposed system compared with other systems using the K-NN method.

Figure 20 illustrates the accumulative distribution function of the estimated localisation errors of the different systems for the K-NN algorithm. This plot also shows the superiority of the proposed system compared to the others. More specifically, the introduced system can achieve a performance that produces localisation errors less than 2.5 m for 80% of cases, while it is around 60% for MODEL II and around 70% for MODEL I. The result that was produced without compensating the BSE outputted the worst, since the body effect errors are not considered when comparing a query fingerprint with the radio map.

**Figure 21:**
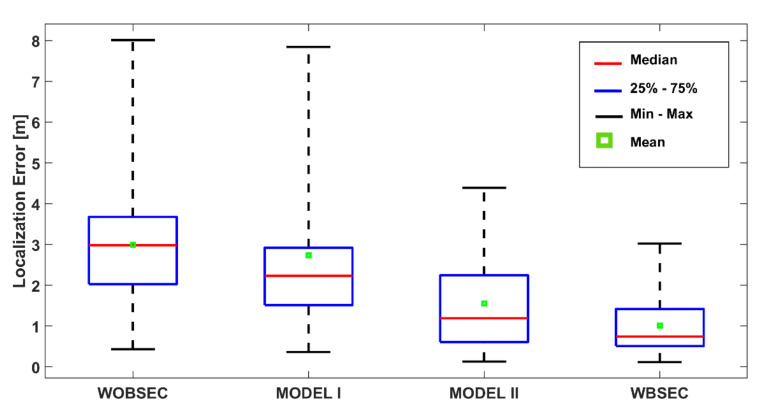
Localisation performance of the proposed system compared with other systems using the WK-NN method.

**Figure 22:**
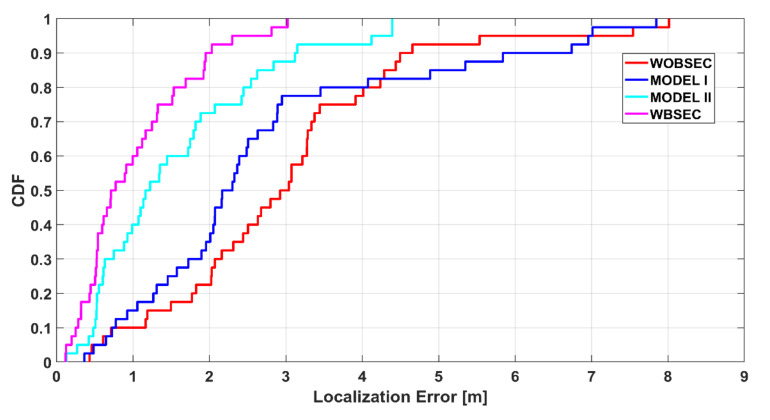
Accumulative distribution of positioning error of the proposed system compared with other systems using the WK-NN method.

The superiority of the proposed system is further increased by applying the WK-NN algorithm when comparing a query fingerprint with the radio map. The performance is improved by including the spatial prominence of the neighbouring RPs in terms of weight factor. Figure 21 and Figure 22, and Table 3 compare the performance of the different systems using the WK-NN algorithm. As can be seen, the WK-NN algorithm with the proposed weighting method improves the localisation accuracy for all the considered systems. The developed system yields a mean error of 1.01 m and median error of 0.74 m, while they are 1.56 m and 1.19 m for MODEL II, and 2.74 m and 2.23 m for MODEL I. The system without any BSE compensation can attain a mean error of 2.99 m and a median error of 2.98 m using the WK-NN algorithms. Moreover, as presented in Figure 22, the WK-NN algorithm produces localisation errors less than 1.5 m for 80% of cases for the proposed system, while it is around 60%, 25%, and 15% for MODEL II, MODEL I, and without applying any compensation, respectively. Overall, the proposed BSE compensation model along with the landmark-assisted WK-NN method is able to achieve sub-metre median localisation accuracy that outperforms some recent related methods.

## 9. Conclusions

This study describes solutions for improving the accuracy of wearable sensor-based fingerprinting indoor localisation by mitigating the user’s BSE on RSSI. An in-depth analysis of RSSI for different orientations of the user’s body was performed, and a body shadowing compensation model is proposed. To calculate the orientation angle between a wearable device and an RN, an IMU-aided motion mode detection technique was implemented by fusing the spatial knowledge from the indoor floor plan. The decision tree-based classifier yields an outstanding performance for motion mode detection that, in turn, accurately identifies the landmark to produce high precision for the estimation of the user’s orientation angle. Results demonstrated that the implemented classifier achieves an overall accuracy of 97.31% for detecting a motion mode correctly, which eventually helps to compensate the errors caused by the user’s body. To validate the proposed body shadowing compensation model, both the classical K-NN and the WK-NN methods were implemented with a unique weighting technique. For selecting the K nearest neighbours in the case of the WK-NN method, the spatial prominence of the neighbouring RPs was applied as the weights, which were calculated by using a unique landmark-assisted distance measurement method. Finally, the localisation performance of the proposed system was compared with two other recent studies that proposed different models for angle estimation and BSE compensation. The experimental results show a mean and median accuracy of 1.62 m and 1.46 m for the classical K-NN method, which is further improved to 1.01 m and 0.74 m, respectively, using the WK-NN method. Overall, the proposed BSE compensation model along with the landmark-assisted WK-NN method can realise sub-metre median localisation accuracy that noticeably outperforms the considered related studies. Although the proposed methods are intended for RSSI fingerprinting localisation, these can be adopted in other RSSI-based indoor localisation applications with body-mounted wearable devices. The main limitation of the proposed system is the dependency of the orientation angle estimation phase on the previously estimated location. Future work will include the implementation of the proposed system in multistorey buildings by addressing this issue.

## Figures and Tables

**Table 2:** Localisation accuracy comparison for different systems with K-NN method.

Method	Min. (m)	Max. (m)	Mean (m)	Median (m)	STD (m)	25th (m)	75th (m)	90th (m)
**WOBSEC**	0.34	9.41	3.17	2.68	1.69	2.10	3.89	5.08
**MODEL I**	0.60	6.46	2.39	1.75	1.64	1.16	3.27	5.60
**MODEL II**	0.38	5.69	2.17	2.38	1.10	1.41	2.74	3.07
**WBSEC**	0.22	4.27	1.62	1.46	0.94	0.92	2.11	2.83

**Table 3:** Localisation accuracy comparison for different systems with the proposed WK-NN method.

Method	Min. (m)	Max. (m)	Mean (m)	Median (m)	STD (m)	25th (m)	75th (m)	90th (m)
**WOBSEC**	0.43	8.01	2.99	2.98	1.65	2.03	3.68	4.58
**MODEL I**	0.36	7.85	2.74	2.23	1.92	1.51	2.92	6.29
**MODEL II**	0.13	4.39	1.56	1.19	1.14	0.61	2.25	3.14
**WBSEC**	0.12	3.02	1.01	0.74	0.73	0.51	1.42	1.99

## References

[1] Li Y., Zhuang Y., Hu X., Gao Z., Hu J., Chen L., He Z., Pei L., Chen K., Wang M. (2021). Toward Location-Enabled IoT (LE-IoT): IoT Positioning Techniques, Error Sources, and Error Mitigation. IEEE Internet Things J..

[2] Wang H., Sen S., Elgohary A., Farid M., Youssef M., Choudhury R.R. No need to war-drive: Unsupervised indoor localization. Proceedings of the 10th International Conference on Mobile Systems, Applications, and Services.

[3] Schmitt S., Adler S., Kyas M. The effects of human body shadowing in RF-based indoor localization. Proceedings of the 2014 International Conference on Indoor Positioning and Indoor Navigation (IPIN).

[4] Popleteev A. (2019). Improving ambient FM indoor localization using multipath-induced amplitude modulation effect: A year-long experiment. Pervasive Mob. Comput..

[5] Bahl P., Padmanabhan V.N. RADAR: An in-building RF-based user location and tracking system. Proceedings of the IEEE INFOCOM 2000.

[6] Gu F., Valaee S., Khoshelham K., Shang J., Zhang R. (2020). Landmark Graph-Based Indoor Localization. IEEE Internet Things J..

[7] Xu C., Firner B., Moore R.S., Zhang Y., Trappe W., Howard R., Zhang F., An N. (2013). SCPL: Indoor device-free multi-subject counting and localization using radio signal strength. Proceedings of the 12th International Conference on Information Processing in Sensor Networks.

[8] Jeong J., Shen Y., Kim S., Choe D., Lee K., Kim Y. (2021). DFC: Device-free human counting through WiFi fine-grained subcarrier information. IET Commun..

[9] Wang Y., Wu K., Ni L.M. (2017). WiFall: Device-Free Fall Detection by Wireless Networks. IEEE Trans. Mob. Comput..

[10] Kianoush S., Savazzi S., Vicentini F., Rampa V., Giussani M. (2017). Device-Free RF Human Body Fall Detection and Localization in Industrial Workplaces. IEEE Internet Things J..

[11] Wang J., Zhang X., Gao Q., Yue H., Wang H. (2017). Device-Free Wireless Localization and Activity Recognition: A Deep Learning Approach. IEEE Trans. Veh. Technol..

[12] Leith D.J., Farrell S. (2020). Coronavirus contact tracing: Evaluating the potential of using bluetooth received signal strength for proximity detection. SIGCOMM Comput. Commun. Rev..

[13] Cully W.P.L., Cotton S.L., Scanlon W.G., McQuiston J.B. Body shadowing mitigation using differentiated LOS/NLOS channel models for RSSI-based Monte Carlo personnel localization. Proceedings of the 2012 IEEE Wireless Communications and Networking Conference (WCNC).

[14] King T., Kopf S., Haenselmann T., Lubberger C., Effelsberg W. COMPASS: A probabilistic indoor positioning system based on 802.11 and digital compasses. Proceedings of the 1st International Workshop on Wireless Network Testbeds, Experimental Evaluation & Characterization.

[15] Della Rosa F., Pelosi M., Nurmi J. (2012). Human-Induced Effects on RSS Ranging Measurements for Cooperative Positioning. Int. J. Navig. Obs..

[16] Trogh J., Plets D., Martens L., Joseph W. Improved Tracking by Mitigating the Influence of the Human Body. Proceedings of the 2015 IEEE Globecom Workshops (GC Wkshps).

[17] Trogh J., Plets D., Thielens A., Martens L., Joseph W. (2016). Enhanced Indoor Location Tracking Through Body Shadowing Compensation. IEEE Sens. J..

[18] Rapiński J., Zinkiewicz D., Stanislawek T. (2016). Influence of human body on Radio Signal Strength Indicator readings in indoor positioning systems. Tech. Sci. Univ. Warm. Mazury Olszt..

[19] Bi J., Wang Y., Li X., Cao H., Qi H., Wang Y. (2018). A novel method of adaptive weighted K-nearest neighbor fingerprint indoor positioning considering user’s orientation. Int. J. Distrib. Sens. Netw..

[20] Deng Z., Fu X., Wang H. (2018). An IMU-Aided Body-Shadowing Error Compensation Method for Indoor Bluetooth Positioning. Sensors.

[21] Plets D., Joseph W., Vanhecke K., Tanghe E., Martens L. (2012). Coverage prediction and optimization algorithms for indoor environments. EURASIP J. Wirel. Commun. Netw..

[22] Wang J., Wang Q. (2013). Body Area Communications: Channel Modeling, Communication Systems, and EMC.

[23] Hall P.S., Hao Y. (2012). Antennas and Propagation for Body-Centric Wireless Communications.

[24] Harmuth H.F., Hussain M.G., Boules R.N. (1999). Electromagnetic Signals: Reflection, Focusing, Distortion, and Their Practical Applications.

[25] Mamun M.A.A., Anaya D.V., Wu F., Redouté J.M., Yuce M.R. Radio Map Building with IEEE 802.15.4 for Indoor Localization Applications. Proceedings of the 2019 IEEE International Conference on Industrial Technology (ICIT).

[26] Ruiz A.R.J., Granja F.S., Honorato J.C.P., Rosas J.I.G. (2012). Accurate Pedestrian Indoor Navigation by Tightly Coupling Foot-Mounted IMU and RFID Measurements. IEEE Trans. Instrum. Meas..

[27] Antigny N., Servières M., Renaudin V. Pedestrian track estimation with handheld monocular camera and inertial-magnetic sensor for urban augmented reality. Proceedings of the 2017 International Conference on Indoor Positioning and Indoor Navigation (IPIN).

[28] Zhang Y., Hu W., Xu W., Wen H., Chou C.T. NaviGlass: Indoor Localisation Using Smart Glasses. Proceedings of the 2016 International Conference on Embedded Wireless Systems and Networks.

[29] Susi M., Renaudin V., Lachapelle G. (2013). Motion Mode Recognition and Step Detection Algorithms for Mobile Phone Users. Sensors.

[30] Kołodziej M., Majkowski A., Tarnowski P., Rak R.J., Gebert D., Sawicki D. (2019). Registration and Analysis of Acceleration Data to Recognize Physical Activity. J. Healthc. Eng..

[31] Reddy S., Mun M., Burke J., Estrin D., Hansen M., Srivastava M. (2010). Using mobile phones to determine transportation modes. ACM Trans. Sen. Netw..

[32] Renaudin V., Susi M., Lachapelle G. (2012). Step Length Estimation Using Handheld Inertial Sensors. Sensors.

[33] Brunato M., Battiti R. (2005). Statistical learning theory for location fingerprinting in wireless LANs. Comput. Netw..

